# The association between influenza infections in primary care and intensive care admissions for severe acute respiratory infection (SARI): A modelling approach

**DOI:** 10.1111/irv.12759

**Published:** 2020-06-12

**Authors:** Liselotte van Asten, Angie Luna Pinzon, Jan van de Kassteele, Gé Donker, Dylan W. de Lange, Dave A. Dongelmans, Nicolette F. de Keizer, Wim van der Hoek

**Affiliations:** ^1^ Centre for Infectious Disease Control Netherlands National Institute for Public Health and the Environment (RIVM) Bilthoven The Netherlands; ^2^ Nivel Primary Care Database – sentinel practices Utrecht the Netherlands; ^3^ National Intensive Care Evaluation Amsterdam the Netherlands; ^4^ Department of Intensive Care Medicine University Medical Center University Utrecht Utrecht the Netherlands; ^5^ Department of Intensive Care Medicine Amsterdam UMC Location AMC Amsterdam The Netherlands; ^6^ Department of Medical Informatics Amsterdam UMC Location AMC Amsterdam Public Health research institute Amsterdam The Netherlands

**Keywords:** association, ILI, influenza, intensive care, pneumonia, regression model, SARI, seasonality, Surveillance, time series, trends

## Abstract

**Background:**

The burden of severe influenza virus infections is poorly known, for which surveillance of severe acute respiratory infection (SARI) is encouraged. Hospitalized SARI patients are however not always tested for influenza virus infection. Thus, to estimate the impact of influenza circulation we studied how influenza in primary care relates to intensive care unit (ICU) admissions using a modelling approach.

**Methods:**

We used time‐series regression modelling to estimate a) the number of SARI admissions to ICU associated with medically attended influenza infections in primary care; b) how this varies by season; and c) the time lag between SARI and influenza time series. We analysed weekly adult ICU admissions (registry data) and adult influenza incidence (primary care surveillance data) from July 2007 through June 2016.

**Results:**

Depending on the year, 0% to 12% of annual SARI admissions were associated with influenza (0‐554 in absolute numbers; population rate: 0/10 000‐0.39/10 000 inhabitants), up to 27% during influenza epidemics. The average optimal fitting lag was +1 week (SARI trend preceding influenza by 1 week), varying between seasons (−1 to +4) with most seasons showing positive lags.

**Conclusion:**

Up to 12% of yearly SARI admissions to adult ICU are associated with influenza, but with large year‐to‐year variation and higher during influenza epidemics. In most years, SARI increases earlier than medically attended influenza infections in the general population. SARI surveillance could thus complement influenza‐like illness surveillance by providing an indication of the season‐specific burden of severe influenza infections and potential early warning of influenza activity and severity.

## 1 INTRODUCTION

Hospital surveillance of severe acute respiratory infection (SARI)^1^ is lacking or incomplete in most Western European countries.^2^, ^3^ Some countries do monitor laboratory‐confirmed influenza hospitalizations or intensive care unit (ICU) admissions and report this to the European Influenza Surveillance Network (EISN).^4^ However, this underestimates severe influenza burden as not all hospital patients admitted with respiratory infections undergo laboratory testing for influenza.^5^ Additionally, denominator data on the number of patients with symptoms of infectious respiratory illness are generally lacking.^3^ The Netherlands, like in most other European countries, has a robust surveillance system for influenza infections in primary care, providing information on timing and duration of the seasonal epidemic.^6^ However, the number of serious complications requiring hospitalization is not available through this system.^5^

In primary care (and other outpatient settings), influenza epidemics are heterogeneous from season to season.^7^, ^8^ This is also reflected by SARI admissions to ICU,^9^ with some seasons showing high peak incidence, while other seasons show lower peaks but sometimes higher cumulative incidence over the season, with or without high ICU mortality.^10^ However, these extremes in ICU do not always coincide with high burden in primary care.^10^ The ratio of SARI in ICU to influenza‐like illness (ILI) in primary care is one of the influenza severity parameters proposed by the World Health Organization (WHO). It expresses the number of SARI ICU admissions per observed ILI patient in primary care.^2^ But, while ILI is the gold standard for estimating influenza activity in the general population, SARI might be less specific for influenza circulation as it could include a higher background level of respiratory disease by other infections and causes.^11^ Thus, to gain better insight into the timing and proportion of SARI ICU admissions that are associated with influenza circulation we used a regression modelling approach.^12^, ^13^ Understanding this association will further elucidate the potential of ICU data for strengthening influenza surveillance.

## 2 METHODS

A long‐running robust ILI surveillance system^6^ and a comprehensive national registry of ICU admissions^14^ provided us with reliable data to make estimates of a) the number of adult SARI ICU admissions associated with medically attended influenza infections in adults in primary care, b) how this varies yearly and c) the delay or lead time between SARI and influenza time series. The study period ran from 1 July 2007 through 30 June 2016. As influenza epidemics occur in winter, we used season‐years which we defined as running from week 27 to 26 (ie approximately from July to June).

### 2.1 Intensive care data

Hospital data on weekly admissions to the ICU were retrieved from the National Intensive Care Evaluation (NICE) registry, originally set up for monitoring quality of ICU care.^14^ As paediatric ICUs are not included in the registry, the study focuses on the adult population. A SARI admission to ICU was defined as a patient meeting all three of the following criteria: (a) the patient was admitted to the hospital less than two days before ICU admission, (b) the ICU admission was not a readmission to the ICU within the hospitalized period, and (c) the APACHE IV^15^ reason for admission was any of the 7 following respiratory codes: *Sepsis, pulmonary; Pneumonia, aspiration; Pneumonia, bacterial; Pneumonia, fungal; Pneumonia, other; Pneumonia, parasitic (ie Pneumocystis pneumonia); and Pneumonia, viral*. Admissions to intensive care for elective surgery or trauma were excluded, and we categorized all remaining admissions as medical admissions. We calculated the proportion of medical ICU admissions that were a SARI by dividing the number of weekly SARI by the weekly number of medical admissions. Information on influenza laboratory testing was not available in the NICE registry. ICU coverage increased during the study period from roughly 40% to near‐complete coverage of all Dutch adult ICUs in 2016.

### 2.2 Influenza‐like Illness data

Medically attended ILI incidence data were retrieved from NIVEL Primary Care Database—sentinel general practitioner (GP) practices. This system covers approximately 0.8% of the Dutch population and is nationally representative for age, sex, regional distribution and population density.^6^ Participating GPs report weekly the number and age of ILI patients. The number of patients registered in their practice was used as a denominator for ILI incidence calculation. To confirm influenza circulation, a subset of ILI patients is systematically swabbed for laboratory testing. We calculated influenza circulation as follows: ILI incidence * the proportion of swabs positive for influenza virus. Influenza epidemics are defined within this ILI surveillance as the weeks with ILI incidence exceeding 5.1/10 000 persons for minimally two consecutive weeks.

### 2.3 Statistical analyses

**Figure 1:**
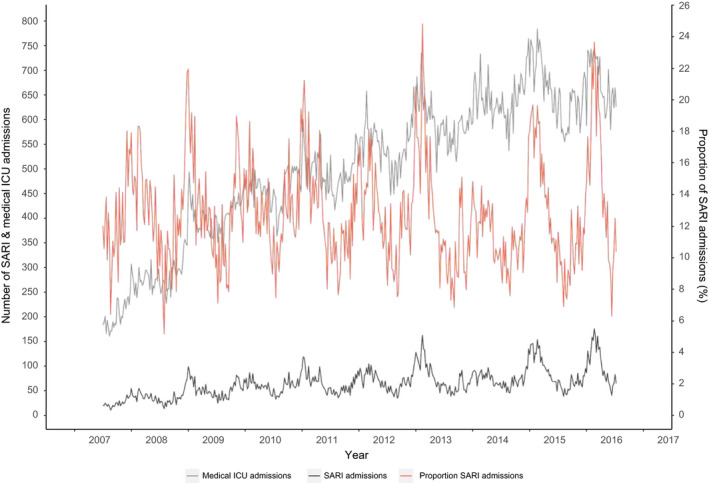
Number of SARI admissions and all medical admissions to adult ICUs

We used a binomial regression model to associate the number of weekly SARI in adult ICU with the weekly influenza incidence in primary care. As the influenza surveillance data contained pre‐defined age groups, we selected the influenza incidence in the 15+ age group as this was the only available age cut‐off for child to adult. The number of SARI admissions $N_{w}^{\text{SARI}}$ in week *w* (*w* = 1, …, 470) was used as outcome variable, while the weekly total number of medical ICU admissions $N_{w}^{\text{ICU}}$ was included in the model as a denominator to adjust for the increasing number of ICUs participating in the NICE registry (Figure 1):

$$N_{w}^{\text{SARI}}∼\text{Binomial}\left(p_{w}^{\text{SARI}},N_{w}^{\text{ICU}}\right).$$

We used an identity link function to relate the proportion of SARI admissions to the explanatory variables, which allows an additive interpretation of the regression coefficients as risk differences instead of odds ratios.

The model for $p_{w}^{\text{SARI}}$ consists of two parts: a) a baseline model for possible underlying seasonal time trends (cyclical) which we assumed to describe SARI admissions associated with other factors than influenza and b) an influenza model that describes the association between the weekly influenza numbers and the number of SARI admissions:

$$p_{w}^{\text{SARI}}=p_{w}^{\text{base}}+p_{w}^{\inf\text{luenza}}.$$

First, we selected the best fitting baseline model consisting of cyclical terms. For the cyclical trend, we evaluated sine and cosine terms with a periodicity of 1, 1/2, 1/3 and 1/4 years. The terms were always included in the model as a sine and cosine pair, thus allowing flexible phase shifts. This resulted in 16 different potential baseline models, always with an intercept, but with and without sine cosine pairs of varying periodicity (ranging from inclusion of zero up to four pairs):

$$p_{w}^{\text{base}}=\beta_{0}+\sum_{k=1}^{4}\left\{\beta_{1k}\sin\left(\frac{2k\pi w}{52.17}\right)+\beta_{1k+1}\cos\left(\frac{2k\pi w}{52.17}\right)\right\}.$$

The model with the lowest Akaike information criterion (AIC) was selected as baseline model. The model for $p_{w}^{\text{influenza}}$ is a bit more complicated, because the dominant influenza strain and severity outcomes (hospitalization, mortality) can vary from season to season.^7^, ^8^, ^10^, ^12^ We therefore performed a time‐dependent analysis. This allowed the association (ie regression coefficient) to vary between seasons. For this, we entered the weekly influenza incidence numbers ${\text{influenza}}_{w}^{s}$ as separate variables per season‐year into the model (being zero everywhere, except for the specific season s). Since we do not know whether SARI ICU admissions follow, coincide or precede the ILI trend, we also evaluated nine lagged values of ILI incidence per season ${\text{ILI influenza}}_{w+j_{s}}^{s}$ (where *j_s_* = −4, …, 4, ie up to 4 weeks earlier and 4 weeks later in time relative to respiratory ICU admissions), including maximally one lag per season. So, per season separately, we added influenza incidence to the baseline model: testing each of the nine ILI lags *j_s_* separately, that is added singularly to the baseline model (thus building nine models per season):

$$p_{w}^{\text{SARI}}=\sum_{s=2007}^{2016}\beta_{s}{\text{influenza}}_{w+j_{s}}^{s}.$$

We repeated this influenza lag selection for each season and per season selected the influenza lag that showed the best fit (lowest AIC).

All analyses were performed using the statistical package R (version 3.4.0). Model selection was performed in this manner, as R would not run all the possible different model fits at once as this produced too many combinations.

We tested both positive and negative lags between influenza and SARI as the direction of this association is still poorly understood, with ICU admissions possibly being earlier.^16^ Influenza circulation may give birth to two distinct populations: vulnerable or fragile persons exposed to influenza in the community may come down with severe illness more quickly than generally healthy persons who may develop ILI symptoms more slowly and/or wait before seeing a GP.

By multiplying the ILI regression coefficients with the observed weekly influenza incidence (lagged according to the season‐specific lags), we calculated the influenza‐associated proportions of SARI (per week). Further multiplying these weekly proportions by the weekly number of medical ICU admissions produced the estimated absolute numbers of weekly SARI associated with influenza. We then cumulated these weekly SARI numbers by season‐year. As the number of ICUs participating in the NICE registry increased over time these absolute numbers were not directly comparable between seasons. Therefore, we chose 2015 as the index year (there was near‐complete national coverage of adult ICUs in NICE) and standardized all estimated numbers to the volume of medical ICU admissions observed in 2015.

## 3 RESULTS

### 3.1 Description of ILI and SARI time series

**Figure 2:**
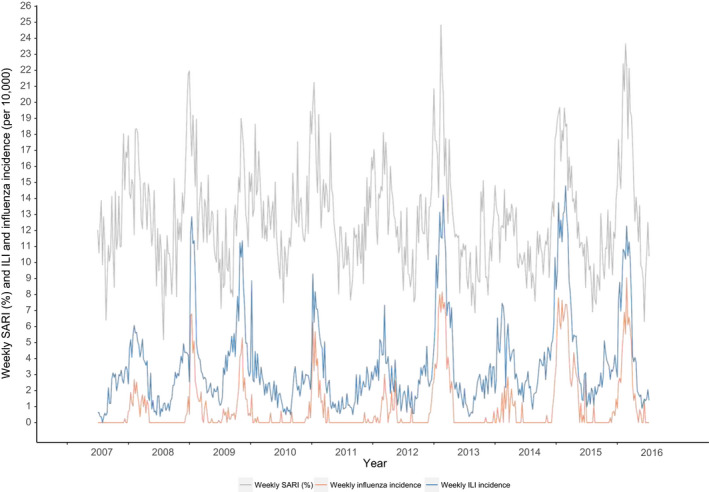
Weekly SARI numbers (as proportion of medical ICU admissions) and weekly ILI and influenza incidence

From July 2007 to June 2016, there were a total of 30 515 registered SARI admissions to ICUs with a weekly average of 65 admissions (standard deviation (SD) 28). To adjust for the increasing coverage of ICUs in the NICE registry, we also show the weekly number of SARI as a proportion (ie relative to weekly total number of medical ICU admissions) (Figure 1). The weekly SARI admissions, ILI and influenza incidence showed peaks in winter with surges roughly coinciding (Figure 2) but with the ILI and influenza trend being more pronounced owing to relatively higher peaks than the trend in SARI. Adult SARI admissions comprised 5% to 25% of medical ICU admissions weekly, ILI incidence varied between 0 and 14.8/10 000, and influenza incidence between 0 and 9.04 /10 000 by week (15+ age group). The correlation coefficient (Spearman's rank) between SARI (proportions) and influenza incidence was 0.60 (*P*‐value < .0001), slightly higher than influenza preceding SARI (0.44‐0.58; lag −4 to −1) or after SARI (0.53‐0.59; lag 1 to 4). Correlations with ILI instead of influenza were similar.

### 3.2 The modelled association between influenza circulation and SARI admissions

**Figure 3:**
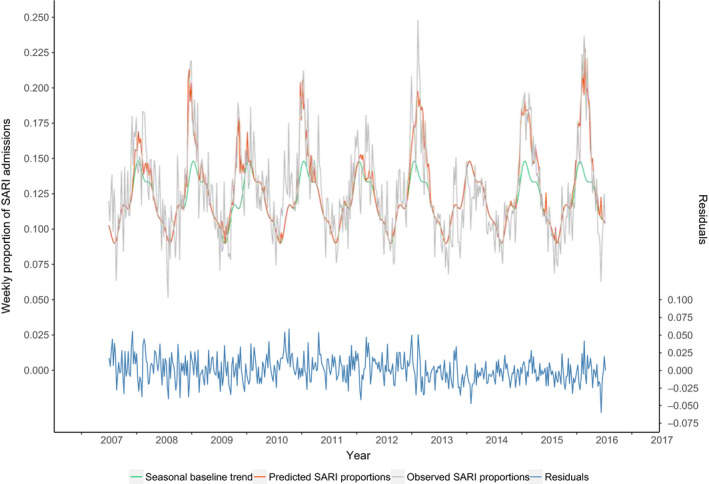
Observed and predicted† weekly proportion of medical admissions due to a SARI (seasons 2007/2008 to 2015/2016). †Predicted weekly proportions were calculated using the parameter estimates from the regression model (with season‐specific estimates)

Observed and modelled SARI numbers are shown in Figure 3. The assumed seasonal baseline (green line) depicts SARI levels the model did not associate with influenza. From the model, we estimated that on average for the total study period, SARI increased with 7.30% (coefficient) with every increase in the influenza incidence of 1/1000 per week (Table 1). For example, if in a certain week the influenza incidence increases with 6/1000 compared to the previous week, the proportion of SARI increases, in an absolute sense, with 6*7.30% = 34.89% compared to the previous week. However, time‐dependent analyses show that this estimate varied significantly from season to season with regression coefficients varying between 0 (season 2013/2014) to 12.13(2009/2010 season) (Table 1).

**Table 1:** Association between respiratory ICU admissions and influenza incidence

Season	Best fitting lag^a^	Coefficient^b^	95% CI	*P*‐value
2007/8 ‐ 2015/16	+1	7.30	6.38 ‐ 8.23	<2.2e‐16
2007/8	+3	7.78	0.79 ‐ 15.05	.030657
2008/9	+4	11.42	8.22 ‐ 14.75	4.59e‐12
2009/10	0	12.13	8.54 ‐ 15.86	1.02e‐10
2010/11	+2	10.37	7.36 ‐ 13.46	2.20e‐11
2011/12	+4	4.06	−1.00 ‐ 9.30	.123481
2012/13	−1	7.06	5.62 ‐ 8.54	<2.2e‐16
2013/14	‐	‐	‐	‐
2014/15	+2	5.74	4.50 ‐ 7.00	<2.2e‐16
2015/16	0	10.03	8.46 ‐ 11.63	<2.2e‐16

^a^ Weeks that SARI admissions are shifted forward (+lags, preceding influenza) or backward (−lags, lagging behind influenza) in time relative to influenza observations.

^b^ Coefficients from a regression analysis representing the proportion of SARI admissions associated with a 1/1000 increase in influenza incidence (adjusted for baseline seasonal trends).

### 3.3 Estimated numbers of SARI associated with influenza

**Figure 4:**
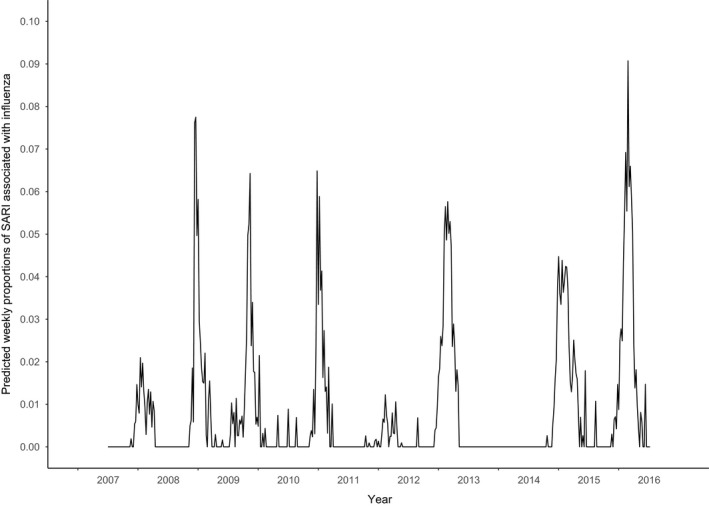
Weekly proportions of SARI associated with influenza. Predicted from a time‐dependent regression model giving season‐specific estimates

**Figure 5:**
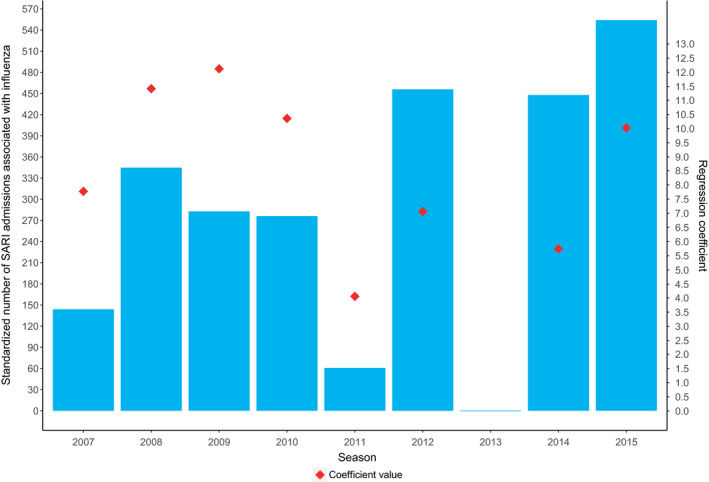
Standardized† number of SARI admissions associated with influenza and regression coefficients per season‡ (2007/8‐2015/16) in the Netherlands. †Standardized to the total number of medical ICU admissions in season 2015/2016. ‡ Each season representing the time period of July until June the next year (eg 2007 representing 2007/2008 season)

On average, 7% of yearly SARI was associated with influenza but with large variations: 0%‐12% of SARI was estimated to be influenza‐associated depending on the season. The highest proportions coincided with the highest absolute number of influenza‐associated SARI (seasons 2012/13, 2014/15 and 2015/16). Figure 4 shows the influenza‐associated proportion per week (depicting the proportion above the cyclical baseline that was estimated to be associated with influenza, ie the value of the red line minus the green line from Figure 3). The estimated absolute number of influenza‐associated SARI in adult ICU (standardized) varied between 0 and 554 for the whole country between the different season‐years (Table 2, Figure 5), on average 321 per season‐year. The 2013/2014 season showed the lowest number (0), the 2015/2016 season showed the highest, followed by the 2012/2013 and 2014/2015 season (554, 456 and 448, respectively, standardized, Table 2, Figure 5). When focusing only on influenza epidemic weeks instead of full season‐years, the percentage of influenza‐associated SARI was higher: on average 18% (0% to 27% between the different epidemics, Table 3). Modelling with ILI instead of influenza showed ILI‐associated SARI to be roughly twofold higher than influenza‐associated SARI in entire season‐years (on average 13% vs 7%), but much less different in influenza epidemic weeks (on average 22% vs 18%).

**Table 2:** SARI admissions and influenza‐associated SARI admissions to ICU per season‐year^a^

	Weekly max of influenza‐associated SARI^b^,	Influenza‐associated SARI (unstandardized)	Influenza‐associated SARI^b^, incidence	Observed crude SARI^b^, incidence	Influenza‐associated SARI^b^, proportion	Influenza‐associated SARI^b^, rate^c^	Observed SARI^b^ rate^c^,
2007/2008	16	52	144	4595	3%	0.10	3.26
2008/2009	52	179	345	4566	8%	0.25	3.24
2009/2010	41	189	283	4408	6%	0.20	3.13
2010/2011	48	202	276	4710	6%	0.20	3.35
2011/2012	9	48	61	4363	1%	0.04	3.10
2012/2013	43	396	456	4680	10%	0.32	3.33
2013/2014	0	0	0	3853	0%	0	2.74
2014/2015	33	451	448	4521	10%	0.32	3.21
2015/2016	66	554	554	4483	12%	0.39	3.19
Yearly average	39	259	321	4464	7%	0.23	3.17

^a^ Full years running from July to June.

^b^ Standardized to the number of medical ICU admissions in season 2015.

^c^ Rates were calculated with total 15+ Dutch population size in 2015 (scale is 1/10 000).

**Table 3:** All SARI admissions and Influenza‐associated SARI admissions during influenza epidemic weeks

	Dominant influenza strains	Influenza vaccine match^a^	% of target groups vaccinated^c^	Epidemic duration (weeks)	Observed crude SARI^b^ incidence (during influenza epidemics)	Influenza‐associated SARI^b^ incidence (during influenza epidemics)	Influenza‐associated SARI^b^ proportion (during influenza epidemics)
2007/2008	A(H1N1) dominance followed by B dominance	mismatch	74%	8	929	58	6%
2008/2009	A(H3N2) dominance	*match*	72%	5	731	88	12%
2009/2010	A(H1N1)pdm09 dominance	mismatch	70%	8	806	178	22%
2010/2011	A(H1N1)pdm09 dominance followed by B dominance	*match*	69%	7	890	153	17%
2011/2012	A(H3N2) dominance	mismatch	66%	0	NA	NA	NA
2012/2013	Mixed A(H1N1)pdm09 and A(H3N2) dominance followed by B dominance	mismatch	62%	16	1996	418	21%
2013/2014	Mixed dominance with slightly more A(H3N2) than A(H1N1)pdm09	mismatch	60%	6	544	0	0%
2014/2015	A(H3N2) dominance followed by B dominance	mismatch	57%	20	2366	404	17%
2015/2016	A(H1N1)pdm09 dominance followed by B dominance	*match*	56%	12	1693	455	27%
2007/2016	Yearly average			9	1244	219	18%

^a^ Vaccine match with the dominant influenza strain(s).

^b^ Standardized to the number of medical ICU admissions in season 2015.

^c^ Individuals aged 60 years or older and individuals with comorbidity who have an increased risk of complications or death due to influenza infection, % as reported previously (Ref 23, 24).

Using Dutch population size numbers of 2015 (as the associated numbers were standardized to year 2015), the absolute numbers translated to the following range of influenza‐associated SARI incidence rates per season‐year: 0/10 000 ‐ 0.39/10 000 (Table 2). Overall raw SARI incidence rates were eight‐ to 78‐fold higher (at 2.7 ‐ 3.4/10 000) than the estimated influenza‐associated SARI rate (Table 2). The peak also varied by season and ranged from a maximum of 0 (2013/2014) to a maximum of 66 (2015/2016) influenza‐associated SARI in one week (standardized, Table 2).

Seasons with higher model coefficients for influenza did not correspond with higher cumulated influenza‐associated SARI (Figure 5) (Spearman's rank *R* ^2^ .12 *P* = .78). Notably, the 2012/2013 and 2014/2015 seasons had an average coefficient size but high total number of influenza‐associated SARI while the 2009/2010 and 2010/2011 season had a high coefficient but an average number of influenza‐associated SARI.

### 3.4 Time lag between SARI and influenza or ILI trends

The overall best fitting influenza lag was on average +1 week for the total study period (ie SARI preceding influenza in the general community by one week showed the best fit). However, the optimal lag varied largely from season to season from −1 to +4 weeks, almost always with positive lags (influenza lagging behind SARI) (Table 1): the best fitting model was achieved when influenza coincided with SARI (lag 0 weeks) in two seasons, lagged behind SARI admissions in five seasons (lag 1 to 4 weeks) and preceded SARI admissions in one season (2012/2013). For the positive lags (SARI preceding influenza), there is no apparent association between the value of the lag and the number of influenza‐associated SARI. When assessing ILI, instead of influenza, the overall best fitting lag was also +1 week (SARI preceding ILI). Four of nine seasons showed similar lags (as between influenza and SARI); however, it was the 2015/2016 season which was the only one in which ILI preceded SARI (lag −2 weeks). This was also the season with the largest proportion of influenza‐associated SARI under study (554, 12%).

## 4 DISCUSSION

This study shows how increases in influenza in primary care relate to increased SARI admissions to adult ICU in time. Varying strongly by season, 0%‐12% (0 to 554) of yearly SARI admissions to ICU are associated with medically attended influenza in the total adult Dutch population, probably reflecting the seasonal variation of circulating influenza strains. In most seasons, increases in ICU admissions occurred 1‐4 weeks earlier than increase of influenza incidence in primary care.

Over the past 10 years, there has been a concerted effort by WHO and ECDC to fill the knowledge gap in our understanding of severe influenza complications requiring hospitalization.^2^ However, a comprehensive SARI surveillance is still lacking in most Western European countries, and how SARI occurrence is associated with influenza circulation is not yet entirely clear. Our study estimates that of all adult SARI admissions to ICU, an overall 7% was associated with influenza as measured by medically attended influenza in the general adult Dutch population. This varied considerably by season‐year (0%‐12%), and was roughly twofold higher when modelling with ILI instead of influenza. The three season‐years with the highest numbers of SARI associated with influenza coincided with the seasons which had the longest influenza epidemics (lasting 12‐20 weeks in 2012/2013, 2014/2015 and 2015/2016).^17^ The percentage of influenza‐associated SARI was higher during influenza epidemic weeks with an average of 18% (0% to 27% between the different epidemics). This is lower than what is found in the extensive sentinel SARI surveillance in Belgium where in 5 influenza epidemics (2013/2014 to 2017/2018) between 31% and 46% of SARI cases were positive for influenza viruses.^1^, ^11^, ^18^ However, direct comparison is difficult as the Belgian estimates are based on all hospitalizations instead of only ICU admissions, with a different SARI case definition than ours, and with a different healthcare system. Few Western European countries other than Belgium have SARI surveillance, although some countries monitor more specifically the number of laboratory‐confirmed influenza hospitalizations and ICU admissions; this narrower case definition shows lower incidences than reported for SARI.^9^, ^19^, ^20^, ^21^

A strength of our study is that we used data from primary care ILI surveillance which is the gold standard for influenza surveillance in the Netherlands and most other Western European countries. Therefore, we assume the model to give the best possible estimate of adult SARI admissions to ICU that are associated with influenza circulation in the general adult population. However, we lacked data to differentiate between influenza cases caused by different types, sub‐types and lineages of circulating influenza viruses. Thus, our estimates provide an average effect in those seasons that multiple influenza viruses played an important role in influenza circulation. There appeared to be no clear association between numbers of influenza‐associated SARI and the dominant circulating influenza virus(es) (Table 3). The associated numbers per season also do not show a straightforward link with seasonal vaccine match or mismatch. Only in three of nine seasons did vaccine match the dominant strain(s), but in those seasons both high (2015/2016) and low (2008/2009) influenza‐associated numbers were apparent (Table 3).^17^, ^22^, ^23^ As influenza vaccination uptake by risk groups (with comorbidities and/or the 60 + age group) has progressively decreased during the study period (from 74% to 56%),^24^, ^25^ the number of SARI associated with influenza has roughly increased over time with two exceptions (2011/2012: no influenza epidemic and 2013/2014: no significant association between influenza and SARI) (table 3).

This study is a population‐level (ecological) study comparing two trends. Although this is currently the best available approach due to a lack of structural laboratory testing of SARI patients for influenza virus, a pitfall in such time‐series analyses is finding associations that may be due to other underlying time trends. To counter this, in the model we included a seasonal baseline assuming that any associations between SARI and other seasonal aspects (for instance climatic factors and other seasonal respiratory pathogens) are accounted for by this baseline. These cyclical terms compete with the influenza variable in the model, which also exhibits a roughly cyclical pattern and the cyclical terms could potentially over‐adjust, leading to an underestimation of the association of interest: between SARI and influenza. Despite this risk of underestimation, we assume the model more valid than when leaving out baseline seasonality because other respiratory pathogens that circulate in autumn and winter can cause severe respiratory symptoms too.^8^ Another risk is posed by potential misclassification of the SARI syndrome, which could also affect the estimation of the number of SARI related to influenza. Excluded diagnoses may lead to underestimation, as in the case of acute exacerbation of chronic obstructive pulmonary disease, since respiratory viruses including influenza viruses are frequently detected in upper and lower respiratory tract samples of such patients.^26^ A final issue warranting further investigation is potential differences by narrower adult age bands. The numbers of ILI patients also swabbed for laboratory influenza diagnosing are small; thus, we analysed the adult population as one whole.

Our data reflect that pressure on ICU is not only defined by the magnitude of the modelled coefficient (the number of SARI expected for every observed influenza) but by the combination with the cumulated incidence of influenza. This is reflected by the 2014/2015 season (with a long 20‐week influenza epidemic) which had an average association with influenza incidence but a high total number of influenza‐associated SARI (448, 10% of SARI), while the 2010/2011 season had a relatively high coefficient but a roughly average number of influenza‐associated SARI. This confirms that multiple measures are required to understand influenza severity and burden in secondary care^10^ and that SARI surveillance would complement ILI surveillance.

Population‐level data as used in this study can provide insight in the timing of trends of SARI admissions relative to medically attended influenza in primary care. While at an individual level a patient is not expected to be admitted to an ICU for an influenza‐associated SARI and thereafter to visit his family physician for ILI, at the population level, trends in severe illness do not necessarily follow trends in mild illness. Literature shedding light on the timing of these trends relative to each other is sparse but reports sometimes suggest that ICU admissions for SARI might actually provide an early indication of severe influenza cases.^16^ In our study, SARI surveillance based on ICU admissions, covering the entire Dutch population, could be a more sensitive measure in detecting influenza epidemic activity than ILI incidence in primary care, which has a more limited population coverage and is highly dependent on health‐seeking behaviour. We previously reported that ILI and SARI data are associated with each other at multiple lag times.^27^ In the current study, we took this further and determined the optimal lead or lag time per season. We saw that timing of the two trends differs greatly from season to season, sometimes coinciding, but more often the SARI trend shows a lead over influenza and ILI trends of 1 to 4 weeks. Only one season (2015/2016, a season of 59% influenza A(H1N1)pdm09 and 39% influenza B in influenza‐positive samples of ILI patients and with reported vaccine match), showed the opposite with SARI lagging 2 weeks behind ILI. This suggests that while SARI in ICU can coincide with ILI trends, ICU admissions more often precede increases in ILI and rarely occur *later* than ILI in primary care, thus being a potentially early indicator for influenza virus activity. SARI surveillance could allow preventive measures and preparedness for increasing pressure on secondary care. This is similar to the finding from a pilot in three Dutch hospitals showing that SARI admissions to hospital (not only in ICU) peaks before ILI (personal communication, S. Marbus, *Hospitals peak first*, submitted 2019). Perhaps vulnerable individuals progress to severe illness more rapidly, or other epidemiological characteristics (eg R0, generation time) may differ from those in the healthier general community. Whether the direction of the time shift is also informative of influenza severity is not clear as only one season in our data showed SARI in ICU lagging behind ILI (by two weeks in 2015/2016, data not shown). That was incidentally also the season with the highest influenza‐associated number and percentage of SARI (or only 2012/2013 in the SARI‐influenza analyses, and second highest). This might suggest that in the other years, when influenza burden on ICU was lower, ICU admittance may have been more accessible leading to earlier admissions and thus explaining the lead times of ICU over primary care. However, of the two seasons with coinciding ILI and SARI trends, one had high numbers (2014/2015) and the other had low numbers (2009/2010) of SARI associated with influenza.

The NICE registry was set up for benchmarking and improving ICU quality. It provides a wealth of data that have potential for additional use. Our results show that it could have additional value for understanding the severity of influenza epidemics. On retrospective data, end‐of‐season estimates can be made as we have done in our current study. Would the data flow be transferred to a more real‐time system—which the registry is aiming to do—it could be used for prospective monitoring of SARI. This would complement the weekly ILI surveillance in primary care and help fill our current knowledge gap on severe influenza complications. Such knowledge is crucial for prevention and response and for estimating the burden and societal cost of influenza epidemics or a pandemic.

## CONFLICT OF INTEREST

L van Asten, AL Luna Pinzon, J. van de Kassteele, G. Donker, DW de Lange, and W. van der Hoek: None to declare. DA Dongelmans is the chairman of the National Intensive Care Evaluation (NICE) registry. NF de Keizer is a board member of the National Intensive Care Evaluation (NICE) registry. NF de Keizer is an employee of the department of medical informatics of the Amsterdam University Medical Center; this department is responsible for processing, maintaining and analysing data of the NICE registry.

## AUTHOR CONTRIBUTION

**Liselotte van Asten:** Conceptualization (equal); Formal analysis (equal); Investigation (equal); Methodology (equal); Writing‐original draft (lead). **Angie Luna Pinzon:** Data curation (equal); Formal analysis (equal); Investigation (equal); Methodology (equal); Writing‐review & editing (equal). **Jan van de Kassteele:** Methodology (equal); Writing‐review & editing (equal). **Gé Donker:** Writing‐review & editing (equal). **Dylan de Lange:** Writing‐review & editing (equal). **Dave A. Dongelmans:** Writing‐review & editing (equal). **Nicolette F. de Keizer:** Writing‐review & editing (equal). **Wim van der Hoek:** Conceptualization (equal); Writing‐review & editing (equal).
